# Biocontrol efficacy of atoxigenic *Aspergillus flavus* strains against aflatoxin contamination in peanut field in Guangdong province, South China

**DOI:** 10.1080/21501203.2021.1978573

**Published:** 2021-10-06

**Authors:** Firew Tafesse Mamo, Bo Shang, Jonathan Nimal Selvaraj, Yongquan Zheng, Yang Liu

**Affiliations:** a School of Food Science and Engineering, Foshan University/South China Food Safety Research Center, Foshan, Guangdong, P R. China; b Key Laboratory of Agro-products Quality and Safety Control in Storage and Transport Process, Ministry of Agriculture and Rural Affairs, Institute of Food Science and Technology, Chinese Academy of Agricultural Sciences, Beijing, P. R. China; c Ethiopian Biotechnology Institute, Addis Ababa, Ethiopia; d The College of Life Sciences, Hubei University, Wuhan, P. R. China; e State Key Laboratory for Biology Pests, Institute of Plant Protection, Chinese Academy of Agricultural Sciences, Beijing, P. R. China

**Keywords:** peanut, Atoxigenic Aspergillus flavusstrains, toxigenic Aspergillus flavusstrains, aflatoxins, biocontrol

## Abstract

Application of atoxigenic strains of *Aspergillus flavus*to soils is the most successful aflatoxin biological control approach. The objective of this study was to evaluate the efficacies of native non-aflatoxin producing (atoxigenic) strains as a biocontrol agent in peanut field in China. The competitive atoxigenic *A. flavus *strains (JS4, SI1and SXN) isolated from different crops, in China were used for field evaluation. The strains applied during the growing season (June – October, 2016) in the field at rate of 25 kg inoculum/hectare. The colonization of these biocontrol agents has been investigated and the population of *A. flavus *communities in soil were determined. The incidences of toxin producing (toxigenic) *A. flavus *strains and aflatoxin contamination in peanuts were also determined. Treated plots produced significant reductions in the incidence of toxigenic isolates of *A. flavus *in soil. However, the total fungal densities were not significantly different (p > 0.05) after treatments. Large percentage of aflatoxin reductions, ranging from 82.8% (SXN) up to 87.2% (JS4) were recorded in treated plots. Generally, the results suggest that the strategy can be used to control aflatoxin contamination and continuous evaluation should be done.

## 1. Introduction

Members of the *Aspergillus* section *Flavi* fungal species (*Aspergillus flavus* and *A.parasiticus*) are known to infect important food crops (Frisvad et al. 2006). Peanut is one of the most vulnerable host crops to *A. flavus* invasion and subsequent aflatoxin (AF) contamination throughout its value chain (Barros et al. 2005). *A. flavus* populations can be subdivided into different groups based on sclerotia size (L-strain with >400 μm in diameter and S-strain <400 μm) (Abbas et al. 2005). Some S-strains produce both AFBs (AFB_1_, AFB_2_) and AFGs (AFG_1_, AFG_2_), whereas others produce only AFBs depending on their geographic origin (Cotty and Cardwell 1999). Other isolates with abundant small sclerotia (diameter <400 µm) classified as strain SBG (Cotty and Cardwell 1999). Within the *A. flavus* population some strains may produce different ranges of AF, called toxigenic, the rest may not produce at all (called atoxigenic) (Donner et al. 2009; B. W. Horn and Dorner 1999). Most of the atoxigenic isolates of *A. flavus* belongs to L-strains. AF are generally carcinogenic (Liu and Wu 2010) immune suppressor (Okoth 2016; Mupunga et al. 2017) as well as cause growth impairments in children (Gong et al. 2002, 2003). AFB_1_ is the most potent and frequently occurring (Kew 2013). Regulatory agencies limit the maximum tolerable limit of AF. The upper limit for AFB_1_ in peanuts is 2 ng/g and 4 ng/g for total AF (B_1_+ B_2_+ G_1_+ G_2_) in the European Union, where as China has a tolerance of 20 ng/g for total AF (FAO 2004), similar limit is adopted by the United States (Wu et al. 2016).

Cyclopiazonic acid (CPA), is an iodole tetramic acid, which was originally discovered in peanuts as a fungal metabolite (Holzapfel 1968). *A. flavus* strains are major producers of CPA, and naturally, they are found to occur as a co-contaminant with AF resulting in important economic losses (Bamba and Sumbali 2005; Astoreca et al. 2014).

The world peanut production totals approximately 42.24 million metric tons in the 2016/2017 growing season, and China was the world’s largest producer contributing to 17 million metric tons (USDA 2017). The five provinces of China such as Guangdong Shandong, Henan, Hebei, and Jiangsu contribute 70% of the country’s production (Yao 2004). Most of the peanut production is located in the South and Southeast regions, where there is relatively higher humidity and temperature favourable for *A. flavus* growth and AF contamination at pre-harvest stage (Cotty and Jaime-Garcia 2007). Reports have revealed higher levels of AF contamination in crops from the southern part of China (like Guangdong province) (Gao et al. 2007; Wu et al. 2016). One strategy that has been developed for reducing preharvest AF contamination of crops is biological control, which is achieved by applying naturally occurring competitive native atoxigenic strains of *A. flavus* to the soil (Horn and Dorner 2009). Atoxigenic *A. flavus* strains interfere with the proliferation of indigenous toxigenic strains (Chang et al. 2012; Pitt et al. 2015; Alaniz Zanon et al. 2016). Also, soil inoculation with atoxigenic strains has a carry-over effect and may protect peanuts from contamination during storage (Dorner and Cole 2002).

Several atoxigenic strains of *A. flavus* have been patented, registered, and commercialised. In USA, from 2004 to 2008 two atoxigenic *A. flavus* strains such as NRRL 21882 (active component of Afla-guard ®) and AF36 (NRRL 18543) were registered and used (Abbas et al. 2011) widely. In addition, a strain K49 (NRRL 30797) has been patented by USDA (King et al. 2011). Several field experiments on the efficacy of potential atoxigenic *A. flavus* have been reported in other parts of the world such as USA, Argentina, Nigeria, Australia, and Thailand (Abbas et al. 2006; Pitt & Hocking 2006; J Atehnkeng et al. 2008; Pitt et al. 2015; Alaniz Zanon et al. 2016). In doing so, significant levels of aflatoxin reduction (43% – 98%) have been achieved.

Interest in the distribution of *A. flavus* species across China has also increased because of increasing suggestions to utilise isolates of atoxigenic strains *A. flavus* to reduce aflatoxin contamination (Yin et al. 2009; Tran-Dinh et al. 2014; Wei et al. 2014; Zhou et al. 2015) Recently, the distribution of *A. flavus* in different agro-ecological zones has been reported (Zhang et al. 2017; Mamo et al. 2018). Molecular characteristics of potential atoxigenic *A. flavus* strains have been reported elsewhere (Jiang et al. 2009; Yin et al. 2009).

Very recently, the efficacy test of atoxigenic *A. flavus* strains against higher aflatoxin producer strains co-inoculated at the equal amount in the soil has been done by group of researchers in China (Yan et al. 2021). In this study, significant aflatoxin reduction (84.96–99.33%) has been achieved. However, no cases were reported before on utilising them in the field condition against the naturally existing multiple fungal strains. Moreover, no cases were reported in China about the carry-on effects of field-applied atoxigenic *A. flavus* on stored peanuts.

Our earlier work has identified 24 potential biocontrol *A. flavus* strains and characterised that all of them were atoxigenic, non-CPA production and lack important aflatoxin biosynthetic genes (from 5 to 17) (Mamo et al. 2018). In the current study, three candidate atoxigenic strains (JS4, SI and SXN) were selected for the field test and they are lack more than 10 AF- biosynthetic genes and two important CPA-biosynthetic genes. Therefore, the efficacy of those three native atoxigenic *A. flavus* strains was evaluated to reduce AF contamination in peanuts under field conditions at the Guangdong province, the southern part of China and their carry-on effects at storage conditions.

## 2. Material and method

### 2.1. Strain selection

**Figure 1.**
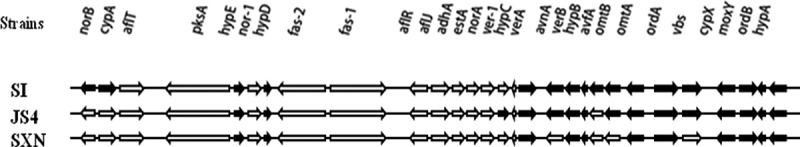
Aflatoxin biosynthesis pathway genes absent patterns of potential biocontrol agents.

**Figure 2.**
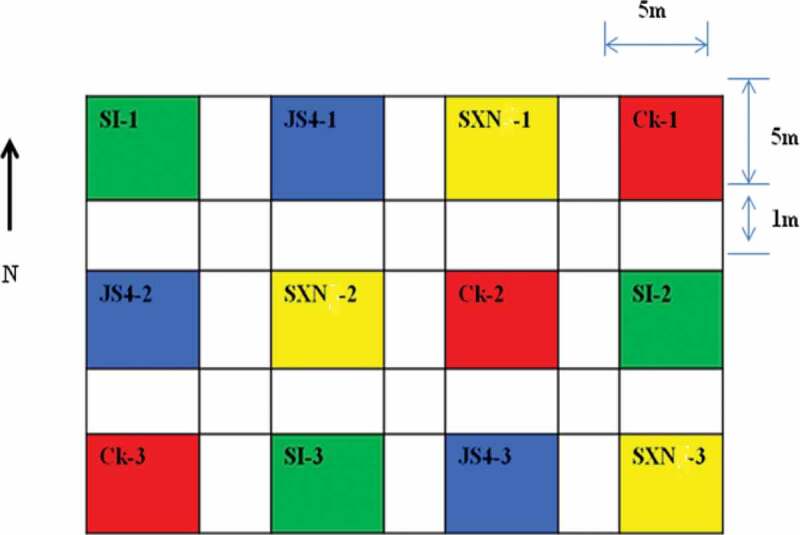
Sketch of the plots for field experiment in Zhanjiang.

The competitive strains used were *A. flavus* strains JS4, SI and SXN. These strains are naturally occurring isolate obtained from crops of China (Table 1). These strains were characterised by Mamoet al.,(2018) and shown to produce neither aflatoxins nor cyclopiazonic acid. The PCR assay revealed that they lost more than 10 aflatoxin biosynthetic cluster genes (Figure 1,2) and none of them did not amplified two functional genes in CPA biosynthesis pathway.

**Table 1.** Information about the competitive atoxigenic *A. flavus* strains used in the field

Strain code	Location	Source	Sclerotia size ^a^	Radial growth rate (cm/day) ^b^	Deletion pattern ^c^
SI	Sichuan	Rice	L	0.67	j
JS4	Jiangsu	Peanut	L	0.64	l
SXN	Shaanxi	Peanut	L	0.62	r

*^a^Sclerotia size: L: sclerotia diameter >400 μm, S: sclerotia diameter <400 μm*

*^b^Radial growth rate (cm/day determined on dilute 1 × 10^4^ CFU/ml) PDA, 3–7 days incubation in darkness at 30 °C. ^C^ Deletion pattern*

### 2.2. Inoculum preparation

The *A. flavus* strain inocula were produced by solid-state fermentation on autoclaved wheat according to (Alaniz Zanon et al. 2016) with little modifications. Briefly, wheat seed (500 g) was soaked in water overnight, drained, placed in a 5-litre flask, and autoclaved. Distilled water was added to attain 35–40% moisture content in the wheat. Starter cultures of *A. flavus* were grown on PDA in 9-cm Petri dishes at 28 °C for 5 days under continuous darkness. The autoclaved wheat was inoculated with 1 ml of a conidial suspension (10^7^/ml), and incubated for 4 days at 30 ° C, the flasks were shaken daily to avoid clump production. At the end of the incubation period, the substrate was dried in a forced-air draft oven at 40°C over night. The dried wheat was then crushed. The viable count (cfu/g) of *A. flavus* in the substrate was determined by homogenising 10 g in 90 ml of peptone water 0.1% (wt/v). This mixture was then shaken and diluted to final concentrations of 10^−2^ and 10^−3^. From each dilution, 0.1 ml was spread in triplicate on Dichloran Rose Bengal Chloramphenicol (DRBC) modified with 3% NaCl (Horn and Dorner, 1999). The Petri dishes were incubated in darkness for 5–7 days at 30°C. All inoculated wheat contained 10^8^cfu *A. flavus*/g.

### 2.3. Field assays

The field assays were done in a commercial field with the previous history of peanut cultivation, located within the peanut-growing region of Guangdong province, Zhanjiang, China. The experiments were established as completely randomised design; the plot consisted of 5 m × 5 m divided into 12 subplots, with a buffer area of 1 m between plots. The peanut cultivar (Zhanyou 75) was planted in rows at 70 cm distance. The planting dates were 24 June 2016. The inoculum was added to fields manually after mixing 62.5 g of pre-inoculated and crushed wheat with 1 kg of sand to attain uniform distribution so that the rate were set at 25 kg inoculum/ha. Inoculation was done on 24 September 2016, 1 month before harvesting (24 October 2016). Subplots comprised controls and treatments as follows: (1) uninoculated control; Ck (2) plots inoculated with atoxigenic *A. flavus* strains JS4, SI and SXN.

### 2.4. Harvest and storage of peanut

Manually harvested peanuts have been dried outside for 7 days and stored in a room in polyethylene bags from October 2016 up to January 2017 for 4 consecutive months. The storage conditions such as relative humidity and temperature were recorded four times a day (6-h interval) and average figures were calculated. At every 30 days of storage time, 3 kg of peanuts were taken to the laboratory of the institute of food science and technology for fungal invasion and aflatoxin B_1_ analysis.

### 2.5. -Soil mycobiota analysis

Soil fungal population analysis was done according to (Alaniz Zanon et al. 2016) with slight modification. Briefly, ten soil samples were taken in two diagonal transects extending from opposing corners in each subplot 1 month before planting and immediately after 15 days of planting and during maturation of the pods prior to digging to determine the soil mycobiota and *Aspergillus* section Flavi (*A. flavus/A. parasiticus*) populations. Each soil sample (approximately 100 g) was a pool from 5 subsamples taken with a trowel from the top 5 cm of soil where peanuts would be or were forming. Sub-samples of each sample were combined in a paper bag and air-dried for 1–2 days at 25–30°C. Samples were thoroughly mixed and passed through a testing sieve (2 mm mesh size).

### 2.6. Soil fungal isolation and identification

From each soil sample 10 g was diluted with 90 ml of peptone water 0.1% (w/v). This mixture was shaken for 20 min and decimally diluted. A 0.1 ml aliquot of each dilution per sample was spread on the surface of solid media: Dichloran Glycerol 18% (DG18). The plates were incubated in darkness for 5–7 days at 30°C. The results were expressed as colony forming units per gram (cfu/g) of soil. Fungal colonies that resembled *Aspergillus* section *Flavi* were sub cultured on malt extract agar medium (MEA) for further identification according to Klich (2002).

### 2.7. Soil toxigenic profile

*A. flavus* isolates were screened for aflatoxin production on coconut cream agar. A modified version of the medium described by (Degola et al. 2011) was used for its efficacy as a diagnostic medium for aflatoxin production: a commercial coconut milk, available from local markets was diluted to 40% (v/v) with distilled water and 15 g agar powder was added, sterilised by autoclaving. Strains inoculated at the centre, after incubation at 30°C for 3 days, media dishes were placed upside down and a drop (0.2 ml) of 25% ammonia solution was placed into the lid of each culture dish to release ammonium vapour (Saito and Machida 1999).Colour development (pink pigmentation) upon contact with ammonium vapour was indicative for aflatoxin synthesis. Consequently, absence of colour development was indicative for absence of aflatoxin development. The plates were scored as positive or negative and isolates were grouped as either toxigenic or atoxigenic (Fani et al. 2014).

### 2.8. Mycobiota analysis from peanut samples

Harvesting and drying were done in October 2016, according to the common practices by the farmers in the area. After drying, four trials of mycobiota analysis were done at each month interval (October 2016 – February 2017). From each subplot, approximately 3 kg of kernels were used. This sample was mixed thoroughly, and 60 kernels (3 replicates) were selected for fungal infection determination. The remaining sample was ground to obtain a subsample of 25 g (3 replicates) for aflatoxin analysis. Peanut kernels from each sub plot were surface disinfected for 1 min in 1% sodium hypochlorite solution, rinsed three times in sterile distilled water and transferred to Petri dishes containing Dichloran Rose Bengal Chloramphenicol agar (DRBC) modified with 3% NaCl (Alaniz Zanon et al. 2016). Plates were incubated at 25°C for 7 days. The incidence of toxigenic isolates of *A. flavus/A. parasiticus* in peanuts was determined by testing all the isolates for toxigenicity as described above for soil samples.

The aflatoxin analysis was performed using the kit following the manufacturer’s instructions (Romer Labs, Getzersdorf, Austria). Briefly described as follows, 25 g of peanuts were ground and put into a 150-ml flask and mixed with 100 ml of the extraction solvent, 84:16 (Acetonitrile: Water, v/v). The mixture was shaken for 60 min at a high speed at 180 rpm in a shaker incubator and filtered with an analytical filter paper. ROMER Aflatoxin clean up column was used for cleaning; 2 ml of the extracted supernatant mixed with 23 ml of phosphate buffer Saline (PBS) modified with 1% tween 20 and passed through the solid-phase extraction column (SPE); on a vacuum manifold at a flow rate of 1–3 drops per second. After column loading, the immune affinity SPE column was washed with 10 ml of PBS before being eluted with 0.5 × 2 ml and 0.5 × 2 ml of water and methanol respectively. The elute was filtered with 0.22 micro litre filter and filled into HPLC vials for HPLC analysis. One hundred micro litres of the extract were injected into the HPLC apparatus (Agilent 2600, Series, Agilent Technology, Germany) with post-column photochemical reactor for enhanced detection (HUAAN, MAGNECH, Beijing, China) with a full loop injection system. The analytical column was an Agilent STC-C1812/250 × 4.6 mm. The column was thermo stated at 30°C. The mobile phase consisted of a mixture of water: methanol (70:30, v/v) eluted at a flow rate of 1.0 ml/ min. The fluorometric detector was set at wavelengths, ex = 365 nm, em = 435 nm. Aflatoxins B_1_, were measured by comparing peak areas with a calibration curves obtained with aflatoxin standard solutions (Sigma-Aldrich, St. Louis, MO, USA). The detection limit was 1ppb.

## 3. Statistical analysis

Completely randomised designs with three to four replicates were used in all experiments. Means separation and comparison were made by Tukey-Kramer HSD test at a probability level of p = 0.05. ANOVA was performed with JMP statistical software (version 13; SAS Institute, Cary, NC). Mean differences in aflatoxin levels of peanut (percent difference between inoculated plot and untreated plots) were calculated as [1 – (total aflatoxin content in peanut from inoculated plots/total aflatoxin content in peanut from untreated plot)] × 100.

## 4. Results

The density of total mycobiota before the application of the bioproducts in the field was homogeneous across all soil samples, with an average count 1 × 10^4^cfu/g. The inoculum levels of native *Aspergillus* section *Flavi* before treatment was also homogenous and similar among plots (1 × 10^2^ cfu/g of soil). *A. flavus* counts were uniform (5 × 10^2^cfu/g) as well.

After treatment, counts of total mycobiota and *Aspergillus s*ection *Flavi* were not significantly different compared with the control plot, both after 15 and 30 days of inoculation (Table 2.). However, a significantly (p < 0.05) higher incidence of (40.8%) of toxigenic *A. flavus* strains was observed in control compared to biocontrol treated plots (Table 2.). Generally, the incidence of toxigenic *A. flavus* decreased in all biocontrol treated plots after 30 days of incubation. All treated plots showed lower incidence of toxigenic *A. flavus* after 30 days of inoculation than 15 days of inoculation. Numerically, JS4 treated plot showed a slightly lower incidence of toxigenic strains in comparison to other treatments both after 15 and 30 days of inoculation (Table 2.).

**Table 2.** Total mycobiota, *Aspergillus* section *Flavi, A. flavus* and incidence of toxigenic *A. flavus* from soil samples before and after treatments

TR	Before treatments				15 days after treatment				30 days after treatment			
	TMB (cfu/g)^a^	ASF (cfu/g)^a^	Af (cfu/g) ^a^	AF+(%) ^b^	TMB (cfu/g) ^a^	ASF (cfu/g)^a^	Af (cfu/g) ^a^	AF+ (%) ^b^	TMB (cfu/g)^a^	ASF (cfu/g)^a^	Af (cfu/g) ^a^	AF+ (%) ^b^
CK	1 × 10^4^	1 × 10^3^	5 × 10^2^	70	1.3 × 10^4^b	1.5x10^3^a	620 c	28.4a	1.6x10^4^a	1.7x10^3^a	250.3b	40.8a
JS4	-	-	-	-	2.6× 10^5^ab	2.7x10^5^a	2.3x10^5^ab	4.9b	2.3x10^5^a	2.1x10^5^a	2.x10^4^a	4.7b
SI	-	-	-	-	1.2× 10^5^ab	7x10^4^a	7x10^4^ab	5.5b	1.7x10^5^a	1.0x10^5^a	1.0x10^4^a	6.1b
SXN	-	-	-	-	1.4× 10^5^ab	7.6x10^4^a	8.3x10^4^ab	12.3ab	1.1x10^5^a	9.6x10^4^a	1.5x10^4^a	4.8b

All the data represent the average values of three replicates.

^a^The counts are expressed as colonies forming units per gram of soil (cfu/g).

^b^These data are expressed as the percentage of the toxigenic strains (AF+(%)).

Abbreviations: TR-refers treatments, CK- control, ASF- *Aspergillus* section *Flavi*, TMB-total mycobiota, Af- *A. flavus* strains

Means in a column followed by the same letter are not significantly different (Tukey-Kramer HSD test, p > 0.05).

At harvesting, there were no significance differences (p> 0.05) both on the peanut infection rate (PIR) and incidence of toxigenic *A. flavus* among all the biocontrol treated plots. Peanut infection rate generally was below 10% in all plots. Across storage time, the incidence of peanut kernels infected with *A. flavus* was higher in treated plots than in the control. PIR shows increment across the storage time from October to January (Table 3). In January, the relatively highest infection rate ranged from 40.0% to 46.1% were seen on the peanuts collected from biocontrol treated plots. However, these values were not significantly different (p> 0.05) (Table 3.). The maximum PIR (35%) by the natural *A. flavus* inoculum was seen in January followed by December (24.4%) (Table 3.). In October and November relatively smaller PIR (10% and 16.1%) were recorded by the natural *A. flavus*. Comparing to control plots, significant reductions in incidence of toxigenic isolates were observed in peanut kernels from biocontrol treated plots across the course of storage time. Stored peanut kernels treated with JS4 show reduced incidence of toxigenic *A. flavus* in December (9.2%) and January (11.8%). On the contrary, the highest 89.9% and 90.7% toxigenic *A. flavus* strains were isolated in peanuts collected from untreated plots during the above storage times, respectively (Table 3.).

**Table 3.** Infection of peanut kernels by total *A. flavus*, percentage of toxigenic *A. flavus* and aflatoxin contamination during the course of storage time

Treatments	October-2016		November-2016		December-2016			January-2017		
	PIR(%)^a^	AF+(%)^b^	PIR(%)^a^	AF+(%)^b^	PIR(%)^a^	AF+(%)^b^	AFB_1_(ppb)^c^	PIR(%)^a^	AF+(%)^b^	AFB_1_(ppb)^c^
Control	10.0bd	77.8a	16.1b	62.5a	24.4b	89.9a	27.3a	35.0a	90.7a	4.2
JS4	23.9ab	13.3b	28.9a	13.4b	37.4ab	9.2 c	3.5 c	41.6a	11.8b	ND
SI	23.3ab	6.5b	32.2a	19.3b	38.3a	12.2 c	4.6b	40.0a	12.0b	ND
SNX_1_	27.2a	10.1b	30.0a	7.2b	37.8ab	19.1b	4.7b	46.1a	21.8b	ND

All the data represent the average values of three replicates.

^a^Peanut infection rate (PIR) is expressed as the percentage of peanut kernels infected with *A. flavus*.

^b^AF+, these data are expressed as the percentage of the toxigenic strains.

^c^Aflatoxin levels (AFB_1_) are expressed as parts per billion (ppb) (ND = not detected; < 1 ppb).

Within a column, values not sharing a common letter are significantly different (Tukey-Kramer HSD test, p < 0:05).

**Figure 3.**
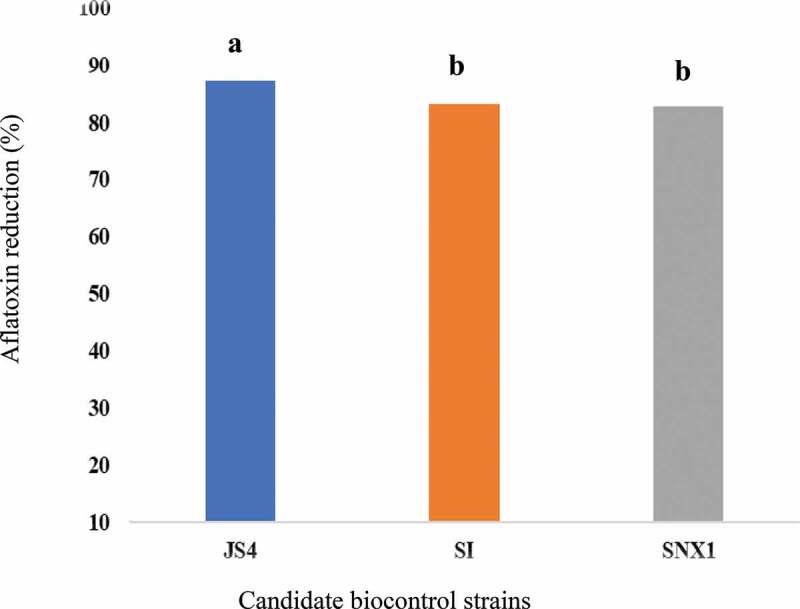
Aflatoxin reduction in peanut kernels harvested.

No AFB_1_ was detected at harvesting time and the first 2 months of storage. It was detected in peanuts in December and January. Significantly (p < 0.005) higher AFB_1_ content (27.3 ppb) was detected in peanut kernels from the untreated plot than biocontrol treated plots at December (Table 3). Significantly lower levels (p < 0.05) AFB_1_ content ranged from 3.5 to 4.7 ppb was detected in peanut kernels from treated plots (Table 3). JS4 treated plot showed the least (3.5 ppb) at this time. In comparison to the uninoculated control plot, large average AFB_1_reduction, ranged from 82.8% (SNX) up to 87.2% (JS4) were recorded (Figure 3). During the last month of storage; in January, no AFB_1_ was detected from all the treatments. However, in average 4.2 ppb AFB_1_ was observed in the control plot (Table 3).

## 5. Discussions

This study provided the first report on the efficacy of *A. flavus* biocontrol strains applied in the peanut field in one of the major peanut-producing regions of China (Guangdong province), Zhejiang. In this field experiment, the native inoculum level (50 cfu/g) and the incidence of toxigenic isolates of *A. flavus* (>50%) were uniform among plots (Average 5 × 10^2^ cfu/g). Similarly, the inoculum level of native *Aspergillus* section *Flavi* in the soil prior to planting was also homogeneous among plots (1 × 10^2^ cfu/g). Among members of *Aspergillus* section *flavi, A. flavus* was the dominant species (93%). These data are consistent with the previous study done in China (Zhang et al. 2017). However, it is lower compared with another study in Argentina (Alaniz et al. 2013).

In the soil samples, no significant changes were observed in the total fungal colony, *Aspergillu*s section *flavi* as well as *A. flavus* counts both after 15 and 30 days of inoculation (Table 2). This implies that, there was no major change on the total fungal population of the field because of the addition of atoxigenic *A. flavus* to the field. Similar results observed in USA (Dorner 2002). Analyses of soils taken prior to harvest showed that the treatments resulted in an increase in the incidence on atoxigenic *A. flavus* in treated plots. The mean *A. flavus* population density in soils before treatment was 5 × 10^2^ cfu/g, of which 70% of isolates tested were toxigenic. The mean *A. flavus* population densities in plots treated with atoxigenic strains at harvest were (JS4, 2 × 10 ^4^ cfu/g), (SI, 1 × 10 ^4^ cfu/g), (SNX, 1 × 10 ^4^ cfu/g) but only 4.0%, 6.1%, and 4.8% of isolates tested were toxigenic for each treatments, respectively. Therefore, application of biocontrol agents had the desired effect of changing the composition of the *A. flavus* soil population to greatly favour the atoxigenic strain.

At harvesting time, peanut infection rate (PIR) by total *A. flavus* population from both treated and untreated plots was lower (<10%) and no detectable aflatoxin observed. The infection rate increased across the storage time for all plots. Relatively, higher peanut infection was recorded in December and January (Table 3.). The infection rate observed at these times (December and January) were not significantly (p> 0.05) different between untreated and treated plots. These data suggest that generally, treatment of soil with atoxigenic strains did not increase total infection of peanuts by *A. flavus*, but rather, it resulted in the preferential invasion of peanuts by atoxigenic strains compared with native toxigenic strain (Dorner and Cole 2002).

At these storage times, the highest incidence (89.9%, 90.7%) of toxigenic *A. flavus* were observed in kernels from the control plot. However, significantly lower incidence of toxigenic (9.2% – 21.8%) *A. flavus* was recorded in kernels from the atoxigenic treated plots. Significant displacement by competitive atoxigenic *A. flavus* strains was occurred. For instance, the strain JS4 showed the highest displacements (from 88.2% to 90.8%) of toxigenic *A. flavus* at the above storage times (Table 3.). This implies that the strain JS4 had profound competitive performance in the field.

**Figure 4.**
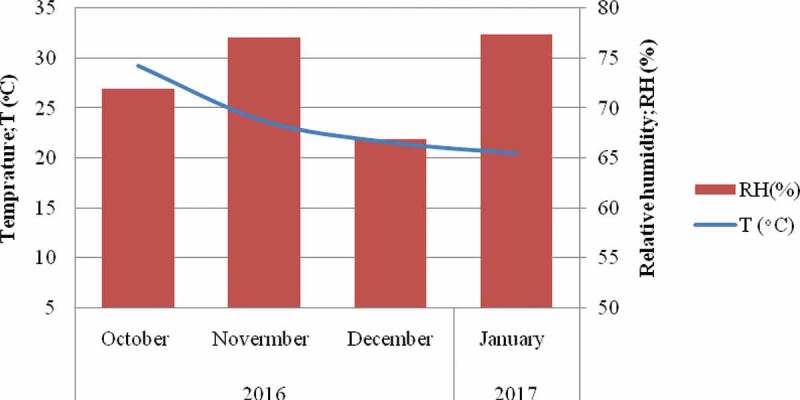
Natural storage conditions of harvested peanuts.

Detectable aflatoxin contamination was not observed at pre-harvest and during the first 2 months of storage time. Delayed aflatoxin contamination is possible because of climatic conditions at pod maturations and natural defence by the peanut kernel. Fungal growth, host invasion and as well as aflatoxin contamination occur when there is optimum environmental condition (Wu et al. 2016). Despite, the mean environmental temperature and humidity registered were 30°C and 79%, respectively. the last period of peanut growth (Figure 4). However, there was continuous rain (266.7 mm) for more than 20 days during this time according to World Weather and Climate Information, data from nearest weather station: Haikou, China (129.8 KM). Furthermore, the natural defence mechanisms of the peanut must be minimised after fungal invasion. Hence, certain periods of time are required for aflatoxin contamination to occur on surface of kernels following penetrating the pod (Diao et al. 2014).

After 2 months of storage AFB_1_ was detected from kernels. Significantly higher AFB_1_ concentration (27.3 ppb) was obtained at December in kernels from untreated plots; this value is by far higher than the national aflatoxin limits (15 ppb) of standards (Wu et al. 2016). However, samples from atoxigenic strain treated plots showed relatively lower (3.5–4.6 ppb) AFB_1_ content at this storage time. This implies that atoxigenic strains reduces AFB_1_ from 82.9% to 87.2%. Similarly, atoxigenic *A. flavus*; JS4 strain demonstrates the highest (87.2%) aflatoxin reduction on storage. Furthermore, except kernels from control plots, no detectable aflatoxin was recorded in January (Table 3). This implies that all atoxigenic (JS4, SI, and SNX) *A. flavus* strains achieved 100% of aflatoxin reduction. However, minimised mean AFB_1_ (4.2 ppb) detected by the native toxigenic leads us as some external environmental factors are attributed to these data.

Overall peanut infection rate of *A. flavus* is 35%. Even though, the atoxigenic *A. flavus* in the control reached to 90.7%, aflatoxin contamination was not higher, this might be attributed to the environmental factors and moreover, the physiology of the crops. A study by Dorner et al. (2003) also revealed as aflatoxin contamination is not always directly correlated with the incidence of invasion by *A. flavus*. Drought, temperature and water stresses are among the environmental factors for aflatoxin contamination to occur (Craufurd et al. 2006). The humidity remains higher (>75%) while the average monthly temperature decreased from 28°C in October to 21°C in January (Figure 4). According to Paterson and Lima (2010, 2011), climate changes (temperature and rainfall) can influence host-pathogen dynamics so does the aflatoxin production. Similarly, the water activity (Aw) that may be reduced across storage time could also possibly affects aflatoxin production on kernels.

## 6. Conclusions

This study proved that field application of indigenous atoxigenic *A. flavus* can efficiently suppress populations of toxigenic strains in the soil and on peanut kernels via the competition and reduce aflatoxin contamination during peanut storage without increasing either the percentage of kernels infected by *A. flavus* in the field or the overall quantity of those fungi present in peanuts after storage. Generally, the competitive exclusion of toxigenic *A. flavus* via atoxigenic ones provides a new strategy for the management of aflatoxin contamination of peanuts and other crops.

## References

[cit0001] Abbas HK, Weaver MA, Zablotowicz RM, Horn BW, Shier WT. 2005. Relationships between aflatoxin production and sclerotia formation among isolates of *Aspergillus* section *Flavi* from the Mississippi Delta. Eur J Plant Pathol. 112(3):283–287.

[cit0002] Abbas HK, Zablotowicz RM, Bruns HA, Abel CA. 2006. Biocontrol of aflatoxin in corn by inoculation with non-aflatoxigenic *Aspergillus flavus* isolates. Biocontrol Sci Technol. 16:437–449. .

[cit0003] Abbas HK, Zablotowicz RM, Horn BW, Phillips NA, Johnson BJ, Jin X, Abel CA. 2011. Comparison of major biocontrol strains of non-aflatoxigenic *Aspergillus flavus* for the reduction of aflatoxins and cyclopiazonic acid in maize. Food Addit Contam. 28(2):198–208. 10.1080/19440049.2010.544680 21259141

[cit0004] Alaniz Zanon MS, Barros GG, Chulze SN. 2016. Non-aflatoxigenic *Aspergillus flavus* as potential biocontrol agents to reduce aflatoxin contamination in peanuts harvested in Northern Argentina. Int J Food Microbiol. 231:63–68. 27220011 10.1016/j.ijfoodmicro.2016.05.016

[cit0005] Alaniz ZMS, Chiotta ML, Giaj-Merlera G, Barros G, Chulze S. 2013. Evaluation of potential biocontrol agent for aflatoxin in Argentinean peanuts. J Food Microbiol. 162(3):220–225. doi:10.1016/j.ijfoodmicro.2013.01.017. 23454811

[cit0006] Astoreca A, Vaamonde G, Dalcero A, Marin S, Ramos A. 2014. Abiotic factors and their interactions influence on the co-production of aflatoxin B1 and cyclopiazonic acid by *Aspergillus flavus* isolated from corn. Food Microbiol. 38:276–283. 24290652 10.1016/j.fm.2013.07.012

[cit0007] Atehnkeng J, Ojiambo PS, Donner M, Ikotun T, Sikora RA, Cotty PJ, Bandyopadhyay R. 2008b. Distribution and toxigenicity of *Aspergillus* species isolated from maize kernels from three agro-ecological zones in Nigeria. Int J Food Microbiol. 122(1–2):74–84. 18180068 10.1016/j.ijfoodmicro.2007.11.062

[cit0008] Atehnkeng J, Ojiambo PS, Ikotun T, Sikora RA, Cotty PJ, Bandyopadhyay R. 2008a. Evaluation of atoxigenic isolates of *Aspergillus flavus* as potential biocontrol agents for aflatoxin in maize. Food Addit Contam Part A Chem Anal Control Expo Risk Assess. 25:1264–1271. 18608502 10.1080/02652030802112635

[cit0009] Bamba R, Sumbali G. 2005. Co-occurrence of aflatoxin B1 and cyclopiazonic acid in sour lime (Citrus aurantifolia Swingle) during post-harvest pathogenesis by *Aspergillus flavus*. Mycopathologia. 159:407–411. 15883727 10.1007/s11046-004-8401-x

[cit0010] Barros G, Torres A, Chulze S. 2005. *Aspergillus flavus* population isolated from soil of Argentina’s peanut-growing region. Sclerotia production and toxigenic profile. J Sci Food Agric. 85(14):2349–2353. doi:10.1002/jsfa.2257.

[cit0011] Chang PK, Abbas HK, Weaver MA, Ehrlich KC, Scharfenstein LL, Cotty PJ. 2012. Identification of genetic defects in the atoxigenic biocontrol strain *Aspergillus flavus* K49 reveals the presence of a competitive recombinant group in field populations. Int J Food Microbiol. 154(3):192–196. 22285533 10.1016/j.ijfoodmicro.2012.01.005

[cit0012] Cotty PJ, Cardwell KF. 1999. Divergence of West African and North American Communities of *Aspergillus* Section *Flavi*. Appl Environ Microbiol. 65(5):2264–2266. 10224034 10.1128/aem.65.5.2264-2266.1999 PMC91331

[cit0013] Cotty PJ, Jaime-Garcia R. 2007. Influences of climate on aflatoxin producing fungi and aflatoxin contamination. Int J Food Microbiol. 119(1–2):109–115. http://linkinghub.elsevier.com/retrieve/pii/S0168160507003959 17881074 10.1016/j.ijfoodmicro.2007.07.060

[cit0014] Craufurd PQ, Prasad PVV, Waliyar F, Taheri A. 2006. Drought, pod yield, pre-harvest *Aspergillus* infection and aflatoxin contamination on peanut in Niger. F Crop Res. 98(1):20–29.

[cit0015] Degola F, Berni E, Restivo FM. 2011. Laboratory tests for assessing efficacy of atoxigenic *Aspergillus flavus* strains as biocontrol agents. Int J Food Microbiol. 146(3):235–243. 21419507 10.1016/j.ijfoodmicro.2011.02.020

[cit0016] Diao E, Dong H, Hou H, Zhang Z, Ji N, Ma W. 2014. Factors influencing aflatoxin contamination in before and after harvest peanuts: a review. J Food Res. 4(1):148–154. http://www.ccsenet.org/journal/index.php/jfr/article/view/43819

[cit0017] Donner M, Atehnkeng J, Sikora RA, Bandyopadhyay R, Cotty PJ. 2009. Distribution of *Aspergillus* section *Flavi* in soils of maize fields in three agroecological zones of Nigeria. Soil Biol Biochem. 41(1):37–44. doi:10.1016/j.soilbio.2008.09.013.

[cit0018] Dorner J, Cole R, Connick W, Daigle D, Mcguire M, Shasha B. 2003. Evaluation of biological control formulations to reduce aflatoxin contamination in peanuts. Biol Control. 26:318–324.

[cit0019] Dorner JW, Cole RJ. 2002. Effect of application of nontoxigenic strains of *Aspergillus flavus* and *A. parasiticus* on subsequent aflatoxin contamination of peanuts in storage. J Stored Prod Res. 38:329–339.

[cit0020] Dorner W 2002. Combined effects of biological control formulations, cultivars, and fungicides on preharvest colonization and aflatoxin contamination of peanuts by *Aspergillus* Species. 79–86.

[cit0021] Fani SR, Moradi M, Probst C, Zamanizadeh HR, Mirabolfathy M, Haidukowski M, Logrieco AF. 2014. A critical evaluation of cultural methods for the identification of atoxigenic *Aspergillus flavus* isolates for aflatoxin mitigation in pistachio orchards of Iran. Eur J Plant Pathol. 140:631–642. .

[cit0022] FAO. 2004. Food and Agriculture Organization (2004) worldwide regulations for mycotoxins in food and feed in 2003. FAO Food and Nutrition Paper 81. Food and Agriculture Organization of the United Nations, Rome, Italy.

[cit0023] Frisvad JC, Thrane U, Samson RA, Pitt JI. 2006. Important mycotoxins and the fungi which produce them. In: Adv Exp Med Biol [Internet]. Vol. 571. [place unknown]; p. 3–31. http://www.ncbi.nlm.nih.gov/pubmed/16408591 10.1007/0-387-28391-9_1 16408591

[cit0024] Gao J, Liu Z, Yu J. 2007. Identification of *Aspergillus* section *Flavi* in maize in northeastern China. Mycopathologia. 164(2):91–95. 17570080 10.1007/s11046-007-9029-4

[cit0025] Gong YY, Cardwell K, Hounsa A, Egal S, Turner PC, Hall AJ, Wild CP. 2002. Dietary aflatoxin exposure and impaired growth in young children from Benin and Togo: cross sectional study. BMJ. 325(7354):20–21. 12098724 10.1136/bmj.325.7354.20 PMC116667

[cit0026] Gong YY, Egal S, Hounsa A, Turner PC, Hall AJ, Cardwell KF, Wild CP. 2003. Determinants of aflatoxin exposure in young children from Benin and Togo, West Africa: the critical role of weaning. Int J Epidemiol. 32(4):556–562. 12913029 10.1093/ije/dyg109

[cit0027] Holzapfel CW. 1968. The isolation and structure of cyclopiazonic acid a toxic metabolite of *Penicillium cyclopium Westling*. Tetrahedron. 24(5):2101–2119. isi:A1968A641100006. 5636916 10.1016/0040-4020(68)88113-x

[cit0028] Horn BW, Dorner JW. 1999. Regional differences in production of aflatoxin B1 and cyclopiazonic acid by soil isolates of *Aspergillus flavus* along a transect within the United States. Appl Environ Microbiol. 65(4):1444–1449. 10103234 10.1128/aem.65.4.1444-1449.1999 PMC91204

[cit0029] Horn BW, Dorner JW. 2009. Effect of nontoxigenic *Aspergillus flavus* and *A. parasiticus* on aflatoxin contamination of wounded peanut seeds inoculated with agricultural soil containing natural fungal populations. Biocontrol Sci Technol. 19(3):249–262.

[cit0030] Jiang J, Yan L, Ma Z. 2009. Molecular characterization of an atoxigenic *Aspergillus flavus* strain AF051. Appl Microbiol Biotechnol. 83:501–505. 19255755 10.1007/s00253-009-1921-z

[cit0031] Kew MC. 2013. A atoxins as a cause of hepatocellular carcinoma. Reviews. 22(3):305–310. 24078988

[cit0032] King ED, Bassi AB, Ross JC, Druebbisch B. 2011. An industry perspective on the use of “atoxigenic” strains of *Aspergillus flavus* as biological control agents and the significance of cyclopiazonic acid. Toxin Rev [Internet]. 30(May):33–41. http://www.pubmedcentral.nih.gov/articlerender.fcgi?artid=3339596&tool=pmcentrez&rendertype=abstract 22844262 10.3109/15569543.2011.588818 PMC3339596

[cit0033] Klich MA. 2002. Identification of common *Aspergillus* species. Netherlands: Centraalbureau voor Schimmelcultures; p. 116.

[cit0034] Liu Y, Wu F. 2010. Global burden of aflatoxin-induced hepatocellular carcinoma: a risk assessment. Environ Health Perspect. 118(6):818–824. 20172840 10.1289/ehp.0901388 PMC2898859

[cit0035] Mamo FT, Shang B, Selvaraj JN, Wang Y, Liu Y. 2018. Isolation and characterization of *Aspergillus flavus* strains in China. J Microbiol. 56(2):1–9. 29392555 10.1007/s12275-018-7144-1

[cit0036] Mupunga I, Mngqawa P, Katerere DR. 2017. Peanuts, aflatoxins and undernutrition in children in Sub-Saharan Africa. Nutrients. 9(12):1–12. 10.3390/nu9121287 PMC5748738 29186859

[cit0037] Okoth S. 2016. Improving the evidence base on aflatoxin contamination and exposure in Africa. Wageningen: The Netherlands. https://www.cta.int/en/issue/.

[cit0038] Paterson RRM, Lima N. 2010. How will climate change affect mycotoxins in food ? Food Res Int [Internet]. 43(7):1902–1914. 10.1016/j.foodres.2009.07.010

[cit0039] Paterson RRM, Lima N. 2011. Further mycotoxin effects from climate change. FRIN [Internet]. 44(9):2555–2566. 10.1016/j.foodres.2011.05.038

[cit0040] Pitt JI, Hocking AD. 2006. Mycotoxins in Australia: biocontrol of aflatoxin in peanuts. Mycopathologia. 162(3):233–243. 16944290 10.1007/s11046-006-0059-0

[cit0041] Pitt JI, Manthong C, Siriacha P, Chotechaunmanirat S, Markwell PJ. 2015. Studies on the biocontrol of aflatoxin in maize in Thailand. Biocontrol Sci Technol. 25(9):1070–1091. doi:10.1080/09583157.2015.1028893.

[cit0042] Saito M, Machida S. 1999. A rapid identification method for aflatoxin-producing strains of *Aspergillus flavus* and *A. parasiticus* by ammonia vapor. Mycoscience [Internet]. 40(2):205–208. doi:10.1007/BF02464300.

[cit0043] Tran-Dinh N, Pitt JI, Markwell PJ. 2014. Selection of nontoxigenic strains of *Aspergillus flavus* for biocontrol of aflatoxins in maize in Thailand. Biocontrol Sci Technol. 3157:1–20. .

[cit0044] USDA USD of A. 2017. World agricultural production.

[cit0045] Wei D, Zhou L, Selvaraj JN, Zhang C, Xing F, Zhao Y, Wang Y, Liu Y. 2014. Molecular characterization of atoxigenic *Aspergillus flavus* isolates collected in China. J Microbiol. 52(7):559–565. 24879349 10.1007/s12275-014-3629-8

[cit0046] Wu L, Ding X, Li P, Du X, Zhou H, Bai Y, Zhang L. 2016. Aflatoxin contamination of peanuts at harvest in China from 2010 to 2013 and its relationship with climatic conditions. Food Control. 60:117–123.

[cit0047] Yan L, Song W, Chen Y, Kang Y, Lei Y, Huai D, Wang Z, Wang X, Liao B. 2021. Effect of non-aflatoxigenic strains of *Aspergillus flavus* on aflatoxin contamination of pre-harvest peanuts in fields in China. Oil Crop Sci. 6(2):81–86.

[cit0048] Yao G 2004. Peanut production and utilization in the People’s Republic of China. Peanut in Local and Global Food Systems Series Report No. 4, USA (Georgia).

[cit0049] Yin Y, Lou T, Yan L, Michailides T, Ma Z. 2009. Molecular characterization of toxigenic and atoxigenic *Aspergillus flavus* isolates, collected from peanut fields in China. J Appl Microbiol. 107(2009):1857–1865. 19457031 10.1111/j.1365-2672.2009.04356.x

[cit0050] Zhang C, Selvaraj JN, Yang Q, Liu Y. 2017. A survey of aflatoxin-producing *Aspergillus* sp. from peanut field soils in four agroecological zones of China. Toxins (Basel). 9(40):1–14. 10.3390/toxins9010040 PMC5308272 28117685

[cit0051] Zhou L, Wei -D-D, Selvaraj JN, Shang B, Zhang C-S, Xing F-G, Zhao Y-J, Wang Y, Liu Y. 2015. A strain of *Aspergillus flavus* from China shows potential as a biocontrol agent for aflatoxin contamination. Biocontrol Sci Technol. 25(5):583–592.

